# Pre-Frailty and Frailty in Dialysis and Pre-Dialysis Patients: A Systematic Review of Clinical and Biochemical Markers

**DOI:** 10.3390/ijerph18189579

**Published:** 2021-09-11

**Authors:** Ana Pereira, Luís Midão, Marta Almada, Elísio Costa

**Affiliations:** 1 Faculty of Medicine, University of Porto, 4200-319 Porto, Portugal; anasrbp@gmail.com; 2 UCIBIO REQUIMTE, Faculty of Pharmacy, Porto4Ageing, University of Porto, 4050-313 Porto, Portugal; luismidao@gmail.com (L.M.); martassalmada@gmail.com (M.A.); 3 School of Medicine and Biomedical Sciences, University of Porto, 4050-313 Porto, Portugal

**Keywords:** frailty, end-stage kidney disease, markers

## Abstract

Patients under dialysis are known to be more vulnerable to frailty, a dynamic geriatric syndrome defined as a state of vulnerability to stressors, due to numerous metabolic changes. With rise of life expectancy globally, it is important to understand the complexity of the pathophysiology of frailty and identify possible markers that can help with the prognosis and diagnosis of frailty. The aim of this systematic review is to give an overview of the knowledge regarding clinical and biochemical markers associated with pre-frailty and frailty in dialysis and pre-dialysis patients. In November 2020, PubMed, Embase and Web of Science were searched. Studies regarding biomarkers associated with (pre-)frailty in (pre-)dialysis patients were included. This systematic review identified clinical and biochemical markers in pre-frail and frail patients under dialysis or pre-dialysis published in the literature. This study shows that more investigation is necessary to identify markers that can differentiate these processes to be used as a diagnostic and prognostic tool in routine care and management of geriatric needs. Interventions that can improve health outcomes in pre-frail and frail older adults under dialysis or pre-dialysis are essential to improve not only the individual’s quality of life but also to reduce the burden to the health systems.

## 1. Introduction

Frailty is a common clinical complication and a state of physical and biological vulnerability mostly diagnosed in the older population. This syndrome is of dynamic nature and is characterized by a progressive decline from robustness to pre-frailty and frailty [1]. The prevalence of frailty and pre-frailty tends to increase with age, and it affects more women compared to men. Frailty predicts more vulnerability to adverse outcomes such as falls, fractures, hospitalization, decreased health-related quality of life and significantly contributes to polypharmacy, increased multimorbidity and mortality [2,3]. In a study involving a vast number of participants with a final sample of 60,816 European community-dwelling individuals from 18 different countries aged 50 years old or over, with the mean age of 67.45 ± 9.71 years, the overall prevalence of pre-frailty was reported as 42.9% and frailty as 7.7%. Significant differences were identified between different countries regarding frailty status [4].

Although the pathophysiology of frailty is not yet clear, it has rather a complex multifactorial etiology characterized by dysregulation of different systems including reduced homeostasis and physiologic reserve, as well as increased vulnerability to poorer health outcomes and mortality. Chronic inflammation, marked by raised serum interleukin 6 (IL-6), C-reactive protein (CRP) and tumor necrosis factor alpha (TNF-α), anemia and lower hematocrit levels, immune activation, as well as obesity and other comorbidities have also been defined as etiologies of frailty [5,6].

Frailty is highly associated with aging, however its high prevalence is also seen in patients with all stages of kidney disease, regardless of their age. It is known that patients suffering from chronic kidney disease (CKD) have an increased predisposition to frailty [7]. Frailty is common in patients with end-stage renal disease (ESRD) and has been reported as affecting two-thirds of these patients [8]. Studies have been carried out to determine the prevalence of frailty amongst dialysis and pre-dialysis patients presenting CKD. In a cohort of 2275 adults undergoing dialysis, the prevalence of frailty was reported as an overall value of 67.7%, going from 44.4% in patients < 40 years old to 78.8% in patients aged 80 or older [9]. In 2012, a cohort studied 336 patients with non-dialysis-dependent CKD and reported prevalence as 14% [10]. A systematic review carried out in 2017 reported the prevalence of frailty from 7% in CKD community-dwellers to 73% in a cohort of patients undergoing hemodialysis. Frailty is an independent risk factor for a series of adverse events in all stages of CKD [11]. In an observational prospective longitudinal study carried out in 2019, a total of 277 patients in dialysis were studied for the presence of frailty. The results were reported as an initial prevalence of 29.6% of frail patients, which increased to 58.5% after a 29-month follow-up. In the same study, a higher risk of mortality was also identified in the frail group compared to the non-frail [12]. Frailty prevalence has also been reported as significantly higher (30–42%) among patients on hemodialysis when compared to the community-dwelling older population. In many other studies, frailty was related to an increased risk of hospitalization and mortality [13].

The literature supports that patients with CKD are, in general, more susceptible to suffering from frailty regardless of age and that the incidence increases with declining kidney function. CKD was classified as a state of accelerated metabolic aging where physiological and biochemical changes can be seen, such as anemia, sarcopenia, reduction of food intake, insulin resistance, persistent inflammation, oxidative stress and osteopenia. Although these changes can be associated with natural aging, it happens more rapidly in patients presenting CKD, leading to premature aging and an earlier manifestation of frailty [14]. Metabolic acidosis, impaired vitamin D metabolism, vascular dysfunction and hyperphosphatemia are other pathophysiological processes present in CKD that can lead to frailty. All these changes associated with the fact that CKD patients have a high prevalence of other comorbidities might be one of the reasons for the high prevalence of frailty in this group [15].

Due to the close association between aging and frailty syndrome, it is important to identify a comprehensive diagnostic tool that can give the information needed to distinguish frail and pre-frail individuals from non-frail. This systematic review aim is to consolidate the information available regarding biomarkers in frail and pre-frail CKD patients undergoing dialysis or in pre-dialysis to identify the biomarkers that can be used as tools to improve efficacy in geriatric medicine practice. The review aims to provide a high level of evidence and to support research groups, policymakers, funding bodies and even reviewers interested in this field by providing these professionals with rapid access to the latest information in patient healthcare management to support their practice and the design of future trials and research programs.

## 2. Materials and Methods

This systematic review was performed following the Cochrane Handbook for Systematic Reviews of Interventions [16]. To ensure that this systematic review addresses a relevant question in a way that benefits the scientific and healthcare community, input was obtained from three people with different and significant backgrounds for the creation of this systematic review, from the identification of the review question until the selection of the final articles. The protocol for this systematic review is registered on PROSPERO under the registration number CRD42020215525.

### 2.1. Review Question

Although there are a few systematic reviews published covering biomarkers in frail patients, it is exceedingly rare to find these studies in ESRD patients. A lack of systematic reviews covering biomarkers in frail and pre-frail patients undergoing dialysis and in pre-dialysis was identified. Therefore, this review aimed to significantly contribute to cover the identified knowledge gap by creating a narrow review question.

The question for this review was created using PICO strategy, following Cochrane guidelines. Therefore, the aim was to gather data from primary articles published referring to biomarkers in pre-frail and frail patients undergoing dialysis and in pre-dialysis.

### 2.2. Inclusion and Exclusion Criteria

The inclusion criteria included primary studies covering clinical and biochemical markers identified in pre-frail and frail patients undergoing dialysis and pre-dialysis.

Articles fulfilling one or more of the exclusion criteria below were excluded during the data selection process for this systematic review:

1. topics unrelated to frailty, dialysis and markers;
2. not data-driven: editorials, letters to editors, opinions, discussion pieces, theoretical papers, reviews, hypothesis;
3. no full text: conference abstracts;
4. not published in English;
5. subjects of the study were not humans;
6. subjects of the study were not on dialysis, not assessed for frailty and no markers were studied.

### 2.3. Data Collection

In November 2020, two reviewers, A.P. and L.M., independently searched and extracted data from PubMed, Embase and Web of Science databases. The final search query was then constructed as: ‘frail* OR geriatric assessment’ AND ‘dialys* OR hemodialys* OR haemodialys* OR ‘end stage kidney disease’ OR ‘chronic kidney disease’ AND ‘marker* OR biomarker*’. Language restrictions were applied to include only studies published in English. No limitations were used on the publication years in the search process. 

**Figure 1:**
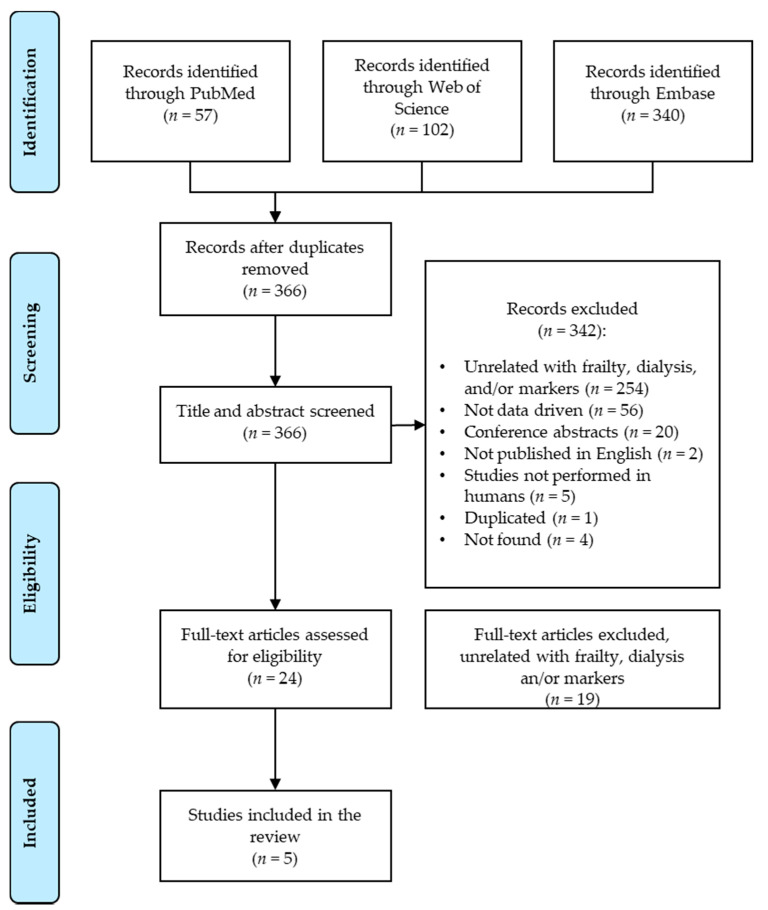
Literature search flowchart—PRISMA format [17].

A total number of 499 articles were found on the three separate databases, which were exported to an Excel spreadsheet and duplicates were removed using Excel tools, leaving a total of 366 articles. The same reviewers independently reviewed the titles and abstracts of all 366 articles and selected the articles with potential interest following the inclusion and exclusion criteria established. Twenty-four articles were selected, and the full text was reviewed for final eligibility evaluating against inclusion and exclusion criteria, selecting a final of five primary studies to be considered for this review (Figure 1). The quality of the final selected articles (*n* = 5) was assessed using a study quality assessment tool for observational cohort and cross-sectional studies from the National Heart, Lung and Blood Institute (NIH).

### 2.4. Interrater Reliability

The data selection process was conducted by two different reviewers, and a third reviewer (M.A.) was used to solve discrepancies. The level of agreement (interrater reliability) among the individuals was measured using SPSS software v.26 (IBM Corp. Armonk, NY, USA). Reliability of data collection is of extreme importance, especially for healthcare and clinical research studies, to ensure a high level of consistency when selecting articles for a systematic review, as well as selecting samples for a study. To measure the interrater reliability of the articles selected for this study, Cohen’s kappa (κ) statistics were used [18].

The selection of articles for this systematic review was done in two phases—selection by title/summary and selection by full article and Cohen’s kappa value obtained for both phases independently.

### 2.5. Ethics

The data used for this systematic review were data already published and publicly available, therefore no ethical approval was undertaken before data collection. Three independent people were part of different stages of the review and Cochrane guidelines were followed to minimize the risk of bias. 

There are no conflict of interests to declare.

## 3. Results

A total number of 499 articles were obtained using the initial search query. After removing 133 duplicates, titles and abstracts of the remaining articles were screened, for a total of 24 articles. Full-text reviews of these articles led to a final selection of five articles (Table 1) meeting the eligibility criteria to be included in the review (Figure 1).

All the studies included in this review were published in 2015 or after (*n* = 5, 100%) with data collected from 2009 onwards, and were carried out in five different countries, representing three continents: America (*n* = 2, 40%), Europe (*n* = 2, 40%) and Asia (*n* = 1, 20%). All the studies included in this review can be broadly classified as observational, however, cohort studies were performed by most of the groups (*n* = 3, 60%) and cross-sectional studies performed by the remaining two groups (40%). The follow-up period in the cohort study carried by one of the studies [19] was 1.1 years, in one other study [20] the population was followed up for 20 months, and 12 months was the follow-up time used by the third group [1]. Most of the studies (*n* = 4, 80%) recruited the population by convenience from the community, although one study [20] did not report the background of the population; contact with the author to obtain information was attempted but no answer was obtained. 

### 3.1. Sample Characteristics

Data were collected from 2009 to 2016; all the studies included adults of both sexes, with a greater percentage of the male population, and a similar age range of >60 years old. Most of the studies (*n* = 4, 80%) had the data collected during the years 2015–2016. Samples size ranges varied from 61 to 605 participants. Three studies (60%) included in this systematic review have samples with more than 100 participants which can be considered significant, however, the studies are not consistent in sample size and there is a considerable gap between the 61-person sample [1] and the study with the biggest sample of 605 people [19]. Some of the studies (*n* = 2, 40%) did not report on ethnicity. In three studies (*n* = 3, 60%), ethnicity was reported as the percentage of non-Caucasian (*n* = 1, 20%), the percentage of African American and Hispanic (*n* = 1, 20%) and the percentage of European, Asian and African (*n* = 1, 20%). The sampling method used was primarily specific population-based cohorts (*n* = 4, 80%) but also convenience sampling (*n* = 1, 20%). A range between 61 to 605 patients, with 60% of the studies (*n* = 3) having a sample size >100, was observed. Regarding dialysis, two of the studies focused on the population undergoing dialysis (40%), two studies (40%) focused on pre-dialysis patients and one study (20%) included both groups.

### 3.2. Exclusion Criteria

Exclusion criteria was used in three studies (60%): patients suffering from specific health conditions were excluded (*n* = 2, 40%), as well as when they were not under dialysis for a specific period of time (*n* = 2, 40%). Excluding patients known to have inflammatory or infectious diseases is essential to avoid the risk of bias. Two studies (40%) did not evaluate sociodemographic parameters, one study (20%) reported on school education, and smoking population percentage was reported in two studies (40%). BMI was reported in two of the studies (40%), while time under dialysis was reported in two studies (40%). Mental state was evaluated in two studies (40%) by the Mini Mental State Examination Scale [23], the Lubben Social Network Scale was measured in one study (20%), while dementia was reported in another different study (20%). Charlson Comorbidity Index was assessed in three studies (60%), with comorbidities and diabetes diagnosis being reported in most of the studies (*n* = 4, 80%). Mansur et al. excluded patients with cognitive impairment from their study.

### 3.3. Biomarkers

In all the studies, biomarkers were measured as continuous variables, however different biomarkers were studied by different groups (Table 1). For easier reading, the biomarkers were grouped as clinical and biological markers in this review. Two studies (40%) reported clinical and biological markers, while the remaining three studies (60%) reported on biological markers only.

Mansur et al. reported a higher level of fat mass and 100% incidence of osteoporosis in frail individuals in pre-dialysis when compared to non-frail. Poveda et al. compared frail to the pre-frail group of patients under dialysis and obtained lower interdialytic weight gain, hemoglobin, transferrin, ferritin and Alb in the frail group. When comparing to the robust group, Poveda et al. obtained lower iron and Alb in the frail group. McAdams-DeMarco et al. studied ESRD patients on the KT list and obtained raised IL-6, sTNFR1 and CRP results in the frail group when comparing to both non-frail and intermediately frail individuals. Ali et al. did not obtain a statically significant difference when compared the results obtained for Hb, Alb, WBC, Ca, PTH and CRP in the frail and non-frail individuals in pre-dialysis. Nakazato et al. compared frail and non-frail groups of patients under dialysis and reported lower results for Alb, Cr and raised LDH and ALP results in the frail group.

### 3.4. Frailty Assessment

Frailty assessment was performed in all the studies included in this review (*n* = 5), however different approaches were taken and the measurement of frailty was performed using different criteria by each study: criteria proposed by Johansen et al. was used by Mansur et al., the FRAIL questionnaire was performed by Poveda et al. and Fried criteria was used by McAdams-DeMarco et al., Ali et al. assessed frailty using a combination of PRISMA questionnaire and Timed Up and Go Test (TUGT) [24], and finally, Nakazato et al. used the Japanese version of the Cardiovascular Health Study Criteria (J-CHS criteria).

### 3.5. Comparison Groups

Regarding comparison groups, three of the studies (*n* = 3, 60%) compared the results of the frail group to a robust/non-frail group, one study (20%) compared the frail group to a robust and a pre-frail group, and the remaining study (*n* = 1, 20%) compared the frail group to a non-frail and intermediately frail group.

## 4. Discussion

Although frailty is a common condition in CKD patients, only recently has it become a topic of interest. No publication date restrictions were applied in this review, yet only five articles were found to meet the inclusion criteria. These were published over the last six years with data collected from 2009 to 2016. On one hand it shows very recent data, on the other hand, the limited number of published articles available hampers comparison and analysis of data from different studies but highlights the opportunity for further investigation. In this review, it was observed that sample characteristics were heterogeneous among articles, as Mansur et al. and Ali et al. reported on pre-dialysis patients and compared frail populations to non-frail; Poveda et al. studied ESRD patients under dialysis and compared frail populations to robust and pre-frail; Nakazato et al. studied dialysis patients and compared frail to non-frail; and McAdams-DeMarco et al. studied ESRD patients and did not differentiate the dialysis from pre-dialysis population and compared frail patients to non-frail and intermediately frail patients. Additionally, the aims of these studies were not specifically to identify biomarkers related to frail and pre-frail patients under dialysis or in pre-dialysis.

Populations from five different countries were studied, however no studies reported on the African or Australian population and most of the studies did not report on ethnicity. Previous studies have reported a higher prevalence of frailty in older persons from ethnic minority groups like African Americans when compared with European Americans, reporting an independent association between African American race and frailty [25]. A recent study performed in the Netherlands reported that older persons with a Turkish, Moroccan and Surinamese background were frailer in comparison with their Dutch background counterparts [26]. Therefore, it is important to investigate differences in the prevalence of frailty between diverse ethnic groups. The majority of the groups carried out cohort studies [1,19,20] with similar follow-up times of 12 to 20 months. However, only mortality, the start of dialysis, hospitalization history and need for renal replacement therapy were variables accounted for at the different follow-ups. It would have been of high interest to measure the clinical and biological markers at the time of the follow-ups. Identification of the number of individuals who progressed from a pre-frail status to a frail status and its correlation with possible biomarker changes would have also been of interest. 

All articles selected for this systematic review recruited their study population by convenience. However, it is known that frailty is more frequent among the female population [4]. Therefore, the sample recruiting method might lead to limitations since most of the studies included in this review reported a higher percentage of male population, which is not an accurate representation of the frail population [4] and might have been a source of bias. 

McAdams-DeMarco et al. in 2018 reported findings in a population ≥ 18 years old, but no average age was reported or was able to be obtained by contacting the authors. However, the average age reported by the remaining studies was > 60 years old, which is aligned with the fact that frailty is often seen in older adults [4]. 

Two of the studies included in this review [1,20] reported markers in the pre-dialysis population, one study [19] focused on ESRD on the kidney transplant list without making a distinction between pre-dialysis and dialysis patients, and the remaining two studies [21,22] reported findings in the dialysis population. Poveda et al. studied the prevalence of frailty in ESRD patients under online-hemodiafiltration (OL-HDF) three times a week for 3–5 h, however no start date of dialysis was considered, and it is known that there are highly fluctuating parameter values during the hemodialysis (HD) initiation phase [27]. Studies have reported differences between CKD pre-dialysis and dialysis patients concerning frailty prevalence and also differences in GFR levels [11].

Making a distinction between pre-dialysis and dialysis patients allows for a more specific investigation and the possibility to analyze the differences independently between both populations. It would have been interesting to measure the biomarkers in a set time from pre-dialysis to dialysis to evaluate the possible changes in the specific markers and their association with frailty.

Poveda et al. reported an association between frailty and lower cognitive function and higher depressive symptoms, which is consistent with studies that found the same association in non-dialysis patients [28]. Poveda et al. have also reported that frail ESRD patients are more susceptible to cognitive impairment and depression due to their exposure to specific factors associated with the dialysis treatment, such as oxidative, inflammatory and hemodynamic stress. A study from 2012 reported an association between frailty and worse cognitive performance and recommended that the frailty assessment should include a cognitive status investigation [29]. 

Mansur et al. observed a high frequency of frailty in pre-dialysis patients and an association with female gender, > 60 years old, endothelial dysfunction and obesity coincident with previous findings [30]. Osteoporosis was found to have an incident of 100% in the frail population in this study. Osteoporosis results in increased bone fragility and accumulated risk of fractures and its prevalence raises alongside with age. Sarcopenia and osteoporosis have been linked and associated with aging, often leading to frailty [31]. Patients with ESRD are more likely to develop osteoporosis and have a higher risk of fractures than their same-age counterparts [32].

Even though vitamin D, CRP, IL-6, TNF-α, Hb and GFR have previously been reported as related to frailty [33], Mansur et al. did not find an association between frailty and these markers, which could be explained by the great variability observed in the results, although the authors identified a strong association between endothelial function and frailty. Ali et al. also studied pre-dialysis patients with a frailty prevalence of 53.8% and did not find significant differences between frail and non-frail groups regarding age, gender, comorbidities, Hb, inflammatory markers or calcium levels. However, they were able to associate frailty with increased mortality, which has also been previously reported in the literature [34]. Peritoneal dialysis was reported by this research group as slightly better in terms of survival when compared to those patients who started on hemodialysis, which is consistent with several studies previously published [35].

Poveda et al. reported on patients under dialysis and hypertension was found to be the etiology of ESRD for the majority of the patients (32.5%), with diabetes for 10.8% and both diabetes and hypertension for 24.1% of the patients. Association was also found between frailty and advanced age, female gender and presence of diabetes and/or hypertension. These findings were expected and consistent with studies in community-dwelling patients [36]. Diabetes and hypertension are the main risk factors for CKD, which can quickly progress to ESRD in patients with uncontrolled diabetes and/or hypertension [37].

Lower interdialytic weight gain, Hb, transferrin and albumin (Alb), and an increase in ferritin serum levels were also reported by Poveda et al. when comparing frail to pre-frail patients. When comparing frail patients to robust patients, lower Fe and Alb was reported.

Hematologic alterations are often seen in ESRD patients under dialysis and a common complication is anemia, which is explained by reduced production of erythropoietin, a renal hormone essential to the production of hemoglobin. Anemia is associated with reduced quality of life, increases morbidity and mortality and accelerates CKD progression. Iron deficiency is also common among CKD patients [38,39]. Low Alb has also been identified to be associated with frailty and increased risk of mortality [40]. Poveda et al. found a correlation between frailty and lower cognitive function, higher prevalence of depression and comorbidities, which agrees with previous studies [28].

McAdams-DeMarco et al. studied ESRD patients on the kidney transplant (KT) waitlist and did not clearly distinguish dialysis from non-dialysis patients. Higher levels of inflammatory markers IL-6, sTNFR1 and CRP have been reported in frail ESRD patients when comparing to both groups of non-frail and intermediately frail individuals. Raised inflammatory markers in frailty are consistent with the literature and have been associated with the pathophysiology of frailty. Raised CRP and IL-6 are correlated with physical disability and muscle weakness. Raised TNFα is associated with mortality in older people [41].

McAdams-DeMarco et al.’s findings support previous studies which report that these inflammatory markers can be used to predict mortality and therefore support the decision to accelerate the care to the patients at greater risk [42].

Nakazato et al. studied patients on HD for longer than six months and found that the frail patients were generally older, had been under HD for a longer period and had lower levels of Alb and Cr and raised LDH and ALP when comparing to non-frail patients.

Lower serum Alb concentrations have been reported as a predictor of higher mortality in HD patients. Hypoalbuminemia has been associated with inflammation, poor nutrient intake and atherosclerotic disease [43]. Decrease in body weight and serum Alb levels have also been reported in HD patients in their final three months of life [44]. Lower Cr levels are common among the older population and have been linked to type 2 diabetes. 

Both Mansur et al. and Ali et al. found a high prevalence of frailty among pre-dialysis patients. Mansur et al. reported these findings in 46% of the < 60 years old. However, while Mansur et al. found an association between frailty, gender, advanced age, obesity and endothelial dysfunction, Ali et al. reported no significant difference when comparing frail and non-frail groups regarding age, gender or any other studied variables. Both studies reported no association between frailty and inflammatory markers, calcium hemostasis and GFR. Ali et al. found no association between frailty and PTH or Alb, while Mansur et al. found no association between vitamin D and frailty. Mansur et al. and Ali et al. reported a link between frailty and pre-dialysis patients and increased risk of mortality.

Nakazato et al. and Poveda et al. both studied patients under dialysis and have both reported frailty as being associated with age. Poveda et al. found a higher prevalence of frail and pre-frail among ESRD under dialysis patients when compared to non-frail. Nakazato et al. also found an association between frailty and duration of HD, low Alb and Cr levels and higher LDH and ALP, while Poveda et al. reported higher prevalence of depressive symptoms, hypertension, lower iron levels, Hb, Alb and transferrin and raised ferritin among frail patients.

No association was found between frailty and Hb or Calcium by Nakazato et al. Despite McAdams-DeMarco et al. not differentiating dialysis from pre-dialysis patients, the group reported 67.7% of the population in study being under dialysis. Higher IL-6, sTNFR1, CRP and inflammatory index were found in frail patients. Inflammation was associated with frailty by this group.

To our knowledge, this is the first systematic review to summarize the information about biomarkers in pre-frail and frail patients in dialysis and pre-dialysis. We were able to identify the biomarkers studied and highlight the ones with statistical significance. 

This systematic review was able to highlight the lack of studies in this area (*n* = 5) and the poor understanding of the etiology of the biomarker alterations due to the complexity of the health conditions presented by these patients. We were, therefore, unable to conclude on the associations between biomarker alterations and their association with frail and pre-frail patients undergoing dialysis or pre-dialysis. The majority of authors did not report on specific biomarkers and their significance but more on the prevalence and clinical outcomes of the conditions. 

Despite the limitations of this review, it does fill a gap in the literature, and it shows the importance of continuing these studies to understand further the association between alteration in biomarkers in these specific patients to allow a better prognostic and care.

## 5. Conclusions

The ambiguities in defining frailty and the complex pathophysiology of this condition makes the development and identification of biomarkers a particularly challenging task. In fact, even the lack of guidelines for selecting a specific tool for assessing frailty is an obstacle.

However, the literature seems to reach consensus when reporting the inflammatory markers of CRP, IL-6 and TNFα. Other biomarkers reported as likely to be related to frailty are Hb, GFR, Alb, hormones such as dehydroepiandrosterone (DHEA) sulfate, testosterone, insulin-like growth factor-a (IGF1) and vitamin D, as well as products of oxidative damage or antioxidants [33].

The inconsistency in approaches noted in the articles included in this review in terms of sample characteristics and biomarkers measured impacts its accuracy and does not offer a robust system able to predict frailty in dialysis patients. The relatively small sample sizes in these studies might have reduced the statistical significance of the biomarkers studied to detect differences between pre-frail and frail and pre-dialysis and dialysis groups.

After analyzing all the articles, it was common to find a high prevalence of frailty in patients under dialysis, particularly in female patients. The higher risk of mortality was also commonly associated with frail patients.

Early identification and intervention are crucial for a potential decrease or even reverse of frailty, especially in the early stages. Routine interventions to try and diminish polypharmacy and review adequacy of medication, nutritional guidance, psychological support and exercise in community-dwelling older adults, especially the ones suffering from CKD under dialysis or pre-dialysis, could be a good and relatively inexpensive strategy to decrease the raising cases of frailty and promote a better quality of life to these patients, improving geriatric care. 

The implementation of this multidimensional approach could be done in the community and primary care centers to minimize poor outcomes such as falls and hospitalization, for example, to reduce the burden on the healthcare services.

A recent report has been published regarding a healthy aging program called ‘HAPPY’ in place in Singapore which aims to engage pre-frail and frail older adults in exercise in the community; an improvement in cognition, physical function, frailty status, reduction of social isolation and improving in perceived health has been reported [45].

Associations between exercise and improved health outcomes in patients suffering from CKD have been reported. A reduction of 22% of mortality among CKD patients under hemodialysis who engage in a 10 min/day physical activity has been reported [46]. Other studies have also reported the importance of light-intensity physical activity among patients suffering from CKD to reduce mortality [47].

Due to the complexity of frail syndrome and the comorbidities frequently related to these patients, including CKD, the identification of biomarkers that can differentiate between frailty and other comorbidities is still in its early stages of the investigation.

However, due to the high prevalence of frailty and the fact that an increase of prevalence is expected in the future as a result of improved life expectance, it is essential that the assessment of frailty is done to identify the early stages of this syndrome and that multidimensional strategies are applied to improve geriatric care, quality of life and reduction in mortality.

## Figures and Tables

**Table 1:** Description of reviewed studies.

First Author, Publication Year	Country, Data Collection Year(s)	Study Design	Sampling Method	Sample Characteristics: Size, Setting, Age, Sex	Exclusion Based on Health Conditions	Biomarkers Measured		Measurement of Frailty	Comparison	Outcome
						Clinical	Biochemical			
Mansur et al. (2015) [1]	Brazil (June 2011–September 2012)	Cohort study	Convenience sampling, CKD patients stages 3–5 on pre-dialysis	61, community, 60 ± 11.5 years old, biological sex: both (59% male)	Severe neuropathy, gout, amputation, severe physical sequelae caused by stroke, DVT, Parkinson’s, pregnancy, COPD, neoplasia, HIV infection, cognitive impairment	BMI Fat mass ^a^ Fat-free mass Abdominal circumference Osteopenia /Osteoporosis ^a^	Creatinine GFR Glycemia TSH Total cholesterol Ferritin TSAT Hb TNF-α IL-6 CRP Ca P Ca × P product Vitamin D iPTH	Johansen et al. criteria	Non-Frail	Frail vs. Non-Frail: Higher fat-mass but not fat-free mass 100% incidence of osteoporosis
Poveda et al. (2017) [21]	Portugal (December 2014–June 2015) ^b^	Cross-sectional study	ESRD patients under dialysis three times a week, for 3–5 h	83, community, average age: 64.3 ± 14.6 years old biological sex: both (53.0% male)	Acute inflammatory or infectious diseases, on dialysis < 3 months, <18 years old	BMI Interdialytic weight gain ^a^	Hb ^a^ RBC MCHC Fe ^a^ Transferrin^a^ Ferritin^a^ Alba	FRAIL scale score	Robust and Pre-Frail	Frail vs. Pre-Frail: . Lower interdialytic weight gain . Lower Hb . Lower transferrin . Lower ferritin . Lower Alb Frail vs Robust: . Lower Fe . Lower Alb
McAdams-DeMarco et al. (2018) [19]	US, (November 2009–May 2016)	Cohort study	ESRD on the KT waiting list, 67.7% in dialysis	605, community, ≥ 18 years old, biological sex: both ^d^	None		CRP ^a^ IL-6 ^a^ sTNFR1 ^a^	Fried criteria	Non-frail and intermediately frail	Frail vs non-frail and intermediately frail: . Higher IL-6 . Higher sTNFR1 . Higher CRP
Ali et al. (2018) [20]	UK (July 2015–July 2016)	Cohort study	Frail and non-frail on pre-dialysis, GFR ≤25 mL	104 ≥ 65 years old, average age 77.1 years (51% male) ^c^	None		Hb Alb WBC Ca PTH CRP	Combination of PRISMA questionnaire and Time up and Go test (TUGT)	Non-frail	Frail vs non-frail: No statistical significance found
Nakazato et al. (2020) [22]	Japan (June 2015–May 2016)	Cross-sectional study	HD patients for >6 months	109, community, average age 63.4 ± 11.3 ^b^, biological sex: both (70.6% male)	Patients on HD for <6 months, patients who had completed <21 of 24 regular blood examinations		LDH ^a^ ALP ^a^ Na AST Cl K UA ALT Plat WBC LDL P HDL Ca TP BUN Cr ^a^ Hb Alb ^a^	Japanese version of the Cardiovascular Health Study criteria (J-CHS criteria)	Non-frail	Frail vs. non-frail: . Lower Alb . Lower Cr . Higher LDH . Higher ALP

^a^*p* < 0.05 is statistically significant. ^b^ Information obtained by contacting author. ^c^ Average age obtained by calculation, as it was unable to be obtained from author. Information about population type was also unable to obtained. ^d^ Unable to obtain information from author regarding average age. Abbreviations: CKD—chronic kidney disease, DVT—deep vein thrombosis, COPD—chronic obstructive pulmonary disease, HIV—human immunodeficiency virus, BMI—body mass index, GFR—glomerular filtration rate, TSH—thyroid-stimulating hormone, TSAT—transferrin saturation, Hb—hemoglobin, TNF-α—tumour necrosis factor alpha, sTNFR1—soluble tumour necrosis factor repector-1, IL-6—interleukin 6, CRP—C-reactive protein, Ca—calcium, P—phosphorus, iPTH—intact parathyroid hormone, PTH—parathyroid hormone, ESRD—end-stage renal disease, RBC—red blood cells, MCHC—mean cell haemoglobin concentration, Fe—Iron, Alb—Albumin, WBC—white blood cells, LDH—lactate dehydrogenase, ALP—alkaline phosphatase, Na—sodium, AST—aspartic aminotransferase, Cl—chloride, K—potassium, UA—uric acid, ALT—alanine aminotransferase, Plat—platelets, LDL—LDL cholesterol, HDL—HDL cholesterol, TP—total protein, BUN—blood urea nitrogen, Cr—creatinine, HD—hemodialysis.

## References

[1] Mansur H.N., Lovisi J.C.M., Colugnati F., Raposo N., Fernandes N., Bastos M.G. (2015). Association of Frailty With Endothelial Dysfunction and Its Possible Impact on Negative Outcomes in Brazilian Predialysis Patients With Chronic Kidney Disease. BMC Nephrol..

[2] Almada M., Brochado P., Portela D. (2020). Prevalence of Falls and Associated Factors Among Community-Dwelling Older Adults: A Cross-Sectional Study. J. Frailty Aging.

[3] Midão L., Brochado P., Almada M., Duarte M., Paúl C., Costa E. (2021). Frailty Status and Polypharmacy Predict All-Cause Mortality in Community Dwelling Older Adults in Europe. Int. J. Environ. Res. Public Health.

[4] Manfredi G., Midão L., Paúl C., Cena C., Duarte M., Costa E. (2019). Prevalence of Frailty Status Among the European Elderly Population: Findings from the Survey of Health, Aging and Retirement in Europe. Geriatr. Gerontol. Int..

[5] Leng S., Chen X., Mao G. (2014). Frailty Syndrome: An Overview. Clin. Interv. Aging.

[6] Leng S., Chaves P., Ms K.K., Walston J. (2002). Serum Interleukin-6 and Hemoglobin As Physiological Correlates in the Geriatric Syndrome of Frailty: A Pilot Study. J. Am. Geriatr. Soc..

[7] Nixon A.C., Bampouras T.M., Pendleton N., Woywodt A., Mitra S., Dhaygude A. (2018). Frailty and Chronic Kidney Disease: Current Evidence and Continuing Uncertainties. Clin. Kidney J..

[8] Worthen G., Tennankore K. (2019). Frailty Screening in Chronic Kidney Disease: Current Perspectives. Int. J. Nephrol. Renov. Dis..

[9] Johansen K.L., Chertow G.M., Jin C., Kutner N.G. (2007). Significance of Frailty Among Dialysis Patients. J. Am. Soc. Nephrol..

[10] Roshanravan B., Khatri M., Robinson-Cohen C., Levin G., Patel K.V., de Boer I.H., Seliger S., Ruzinski J., Himmelfarb J., Kestenbaum B. (2012). A Prospective Study of Frailty in Nephrology-Referred Patients With CKD. Am. J. Kidney Dis..

[11] Chowdhury R., Peel N., Krosch M., Hubbard R. (2017). Frailty and Chronic Kidney Disease: A Systematic Review. Arch. Gerontol. Geriatr..

[12] Garcia-Canton C., Rodenas A., Lopez-Aperador C., Rivero Y., Anton G., Monzon T., Diaz N., Vega N., Loro J., Santana A. (2019). Frailty in Hemodialysis and Prediction of Poor Short-Term Outcome: Mortality, Hospitalization and Visits to Hospital Emergency Services. Ren. Fail..

[13] Johansen K.L., Dalrymple L.S., Delgado C., Chertow G.M., Segal M.R., Chiang J., Grimes B., Kaysen G.A. (2017). Factors Associated With Frailty and Its Trajectory Among Patients on Hemodialysis. Clin. J. Am. Soc. Nephrol..

[14] Kuningas K., Inston N. (2021). Age Is Just a Number: Is Frailty Being Ignored in Vascular Access Planning for Dialysis?. J. Vasc. Access.

[15] Smith G., Avenell A., Band M.M., Hampson G., Lamb E.J., Littleford R.C., McNamee P., Soiza R.L., Sumukadas D., Witham M.D. (2021). Associations Between Frailty, Physical Performance, and Renal Biomarkers in Older People With Advanced Chronic Kidney Disease. Eur. Geriatr. Med..

[16] Higgins J.P.T., Thomas J., Chandler J., Cumpston M., Li T., Page M.J., Welch V.A. (2021). Cochrane Handbook for Systematic Reviews of Interventions version 6.2 (updated February 2021). Cochrane. www.training.cochrane.org/handbook.

[17] Cohen J. (1960). A Coefficient of Agreement for Nominal Scales. Educ. Psychol. Meas..

[18] McAdams-DeMarco M.A., Ying H., Thomas A., Warsame F., Shaffer A., Haugen C.E., Garonzik-Wang J.M., Desai N.M., Varadhan R., Walston J. (2018). Frailty, Inflammatory Markers, and Waitlist Mortality Among Patients With End-Stage Renal Disease in a Prospective Cohort Study. Transplantation.

[19] Baharani J., Ali H., Abdelaziz T., Abdelaal F. (2018). Assessment of Prevalence and Clinical Outcome of Frailty in an Elderly Predialysis Cohort Using Simple Tools. Saudi J. Kidney Dis. Transplant..

[20] Folstein M.F., Folstein S.E., McHugh P.R. (1975). “Mini-mental state”: A practical method for grading the cognitive state of patients for the clinician. J. Psychiatr. Res..

[21] Raîche M., Hébert R., Dubois M.-F. (2008). PRISMA-7: A Case-Finding Tool to Identify Older Adults With Moderate to Severe Disabilities. Arch. Gerontol. Geriatr..

[22] Moher D., Liberati A., Tetzlaff J., Altman D.G. (2009). Preferred reporting items for systematic reviews and meta-analyses: The PRISMA statement. BMJ.

[23] Hirsch C., Anderson M.L., Newman A.B., Kop W., Jackson S., Gottdiener J., Tracy R., Fried L.P. (2006). The Association of Race With Frailty: The Cardiovascular Health Study. Ann. Epidemiology.

[24] Franse C.B., Van Grieken A., Qin L., Melis R.J.F., Rietjens J.A.C., Raat H. (2018). Ethnic Differences in Frailty: A Cross-Sectional Study of Pooled Data from Community-Dwelling Older Persons in the Netherlands. BMJ Open.

[25] Nakazato Y., Sugiyama T., Ohno R., Shimoyama H., Leung D.L., Cohen A.A., Kurane R., Hirose S., Watanabe A., Shimoyama H. (2020). Estimation of Homeostatic Dysregulation and Frailty Using Biomarker Variability: A Principal Component Analysis of Hemodialysis Patients. Sci. Rep..

[26] Poveda V., Filgueiras M., Miranda V., Santos-Silva A., Paúl C., Costa E. (2017). Frailty in End-Stage Renal Disease Patients under Dialysis and Its Association With Clinical and Biochemical Markers. J Frailty Aging.

[27] Nakazato Y., Kurane R., Hirose S., Watanabe A., Shimoyama H. (2017). Aging and Death-Associated Changes in Serum Albumin Variability over the Course of Chronic Hemodialysis Treatment. PLoS ONE.

[28] Searle S.D., Rockwood K. (2015). Frailty and the Risk of Cognitive Impairment. Alzheimer’s Res. Ther..

[29] Macuco C.R.M., Batistoni S., Lopes A., Cachioni M., Falcão D., Neri A.L., Yassuda M.S. (2012). Mini-Mental State Examination Performance in Frail, Pre-Frail, and Non-Frail Community Dwelling Older Adults in Ermelino Matarazzo, São Paulo, Brazil. Int. Psychogeriatrics.

[30] Blaum C.S., Xue Q.L., Michelon E., Semba R.D., Fried L.P. (2005). The Association Between Obesity and the Frailty Syndrome in Older Women: The Women’s Health and Aging Studies. J. Am. Geriatr. Soc..

[31] Greco E.A., Pietschmann P., Migliaccio S. (2019). Osteoporosis and Sarcopenia Increase Frailty Syndrome in the Elderly. Front. Endocrinol..

[32] Alem A.M., Sherrard D.J., Gillen D.L., Weiss N.S., Beresford S.A., Heckbert S.R., Wong C., Stehman-Breen C. (2000). Increased Risk of Hip Fracture Among Patients With End-Stage Renal Disease. Kidney Int..

[33] Al Saedi A., Feehan J., Phu S., Duque G. (2019). Current and Emerging Biomarkers of Frailty in the Elderly. Clin. Interv. Aging.

[34] Kojima G., Iliffe S., Walters K. (2018). Frailty Index As a Predictor of Mortality: A Systematic Review and Meta-Analysis. Age Ageing.

[35] Sachdeva B., Zulfiqar H., Aeddula N.R. Peritoneal Dialysis. https://www.ncbi.nlm.nih.gov/books/NBK532979/.

[36] Fried L.P., Tangen C.M., Walston J., Newman A.B., Hirsch C., Gottdiener J., Seeman T., Tracy R., Kop W.J., Burke G. (2001). Frailty in Older Adults: Evidence for a Phenotype. J. Gerontol. Ser. A Biol. Sci. Med. Sci..

[37] Kazancioğlu R. (2013). Risk Factors for Chronic Kidney Disease: An Update. Kidney Int. Suppl..

[38] Gluba-Brzózka A., Franczyk B., Olszewski R., Rysz J. (2020). The Influence of Inflammation on Anemia in CKD Patients. Int. J. Mol. Sci..

[39] Lankhorst C.E., Wish J.B. (2010). Anemia in Renal Disease: Diagnosis and Management. Blood Rev..

[40] Yanagita I., Fujihara Y., Iwaya C., Kitajima Y., Tajima M., Honda M., Teruya Y., Asakawa H., Ito T., Eda T. (2020). Low Serum Albumin, Aspartate Aminotransferase, and Body Mass Are Risk Factors for Frailty in Elderly People With diabetes–a Cross-Sectional Study. BMC Geriatr..

[41] Hubbard R.E., O’Mahony M.S., Savva G., Calver B.L., Woodhouse K.W. (2009). Inflammation and Frailty Measures in Older People. J. Cell. Mol. Med..

[42] Bao Y., Dalrymple L., Chertow G.M., Kaysen G.A., Johansen K.L. (2012). Frailty, Dialysis Initiation, and Mortality in End-Stage Renal Disease. Arch. Intern. Med..

[43] Leavey S.F., Strawderman R.L., Young E.W., Saran R., Roys E., Agodoa L.Y., Wolfe R.A., Port F.K. (2000). Cross-Sectional and Longitudinal Predictors of Serum Albumin in Hemodialysis Patients. Kidney Int..

[44] Kotanko P., Thijssen S., Usvyat L., Tashman A., Kruse A., Huber C., Levin N.W. (2009). Temporal Evolution of Clinical Parameters before Death in Dialysis Patients: A New Concept. Blood Purif..

[45] Merchant R.A., Tsoi C.T., Tan W.M., Lau W., Sandrasageran S., Arai H. (2021). Community-Based Peer-Led Intervention for Healthy Ageing and Evaluation of the ‘HAPPY’ Program. J. Nutr. Heal. Aging.

[46] Matsuzawa R., Matsunaga A., Wang G., Kutsuna T., Ishii A., Abe Y., Takagi Y., Yoshida A., Takahira N. (2012). Habitual Physical Activity Measured by Accelerometer and Survival in Maintenance Hemodialysis Patients. Clin. J. Am. Soc. Nephrol..

[47] Beddhu S., Wei G., Marcus R.L., Chonchol M., Greene T. (2015). Light-Intensity Physical Activities and Mortality in the United States General Population and CKD Subpopulation. Clin. J. Am. Soc. Nephrol..

